# Adrenal Aging and Its Implications on Stress Responsiveness in Humans

**DOI:** 10.3389/fendo.2019.00054

**Published:** 2019-02-07

**Authors:** Andreas Yiallouris, Constantinos Tsioutis, Eirini Agapidaki, Maria Zafeiri, Aris P. Agouridis, Dimitrios Ntourakis, Elizabeth O. Johnson

**Affiliations:** ^1^School of Medicine, European University Cyprus, Nicosia, Cyprus; ^2^Laboratory of Education & Research Neuroscience, Department of Anatomy, School of Medicine, National and Kapodistrian University Athens, Athens, Greece; ^3^Society of Junior Doctors, Athens, Greece; ^4^Diabetes and Obesity Center, Konstantopouleio Hospital, Athens, Greece

**Keywords:** senescence, adrenal cortex, stress, HPA axis, glucocorticoids

## Abstract

Normal aging results in subtle changes both in ACTH and cortisol secretion. Most notable is the general increase in mean daily serum cortisol levels in the elderly, without a noteworthy alteration in the normal circadian rhythm pattern. Glucocorticoid excess seen in the elderly population can have serious consequences in both the structural and functional integrity of various key areas in the brain, including the hippocampus, amygdala, prefrontal cortex, with consequent impairment in normal memory, cognitive function, and sleep cycles. The chronically elevated glucocorticoid levels also impinge on the normal stress response in the elderly, leading to an impaired ability to recover from stressful stimuli. In addition to the effects on the brain, glucocorticoid excess is associated with other age-related changes, including loss of muscle mass, hypertension, osteopenia, visceral obesity, and diabetes, among others. In contrast to the increase in glucocorticoid levels, other adrenocortical hormones, particularly serum aldosterone and DHEA (the precursor to androgens and estrogens) show significant decreases in the elderly. The underlying mechanisms for their decrease remain unclear. While the adrenomedullary hormone, norephinephrine, shows an increase in plasma levels, associated with a decrease in clearance, no notable changes observed in plasma epinephrine levels in the elderly. The multiplicity and complexity of the adrenal hormone changes observed throughout the normal aging process, suggests that age-related alterations in cellular growth, differentiation, and senescence specific to the adrenal gland must also be considered.

## Introduction

Normal aging is associated with multi endocrine changes, including those associated with changes in the structure and function of the adrenal gland. The various morphological changes of the adrenal gland that occur during aging are associated with alterations in hormonal output, such as a gradual sustained, increase in glucocorticoid secretion and decline in adrenal androgen levels. The increase in circulating levels of cortisol in aging individuals is of particular interest due to the impact of cortisol on several systems, including cognition, and the inherent relationship of chronic stress, elevated cortisol, and aging.

Stress is a constant factor in modern life. The stress response in healthy organisms is aimed at maintaining the balance of biological functions, or homeostasis, when faced with physiological or psychological challenges, that may be real or even perceived. The normal stress response entails a tight orchestration of several adaptive response cascades of the central nervous system and the neuroendocrine systems that are targeted at facilitating homeostasis and ultimately, survival. An integral part of the response entails activation of stress neural circuits, which link brain regions responsible for basic sensory and motor functions for perception and motor response to the stressful challenge, respectively, as well as more intricate autonomic, neuroendocrine, cognitive, and behavioral activities. While activation of these neural circuits is considered part of the normal stress response, chronic stress may deregulate these circuits and responses, resulting in impaired function of these systems.

The stress response system is comprised of central and peripheral components. Of these, the hypothalmic-pituitary-adrenal (HPA) axis has been defined as a primary player in the stress response. The HPA axis has been the subject of intense basic and clinical research in the attempt to understand why the primary adrenal hormonal output, glucocorticoids, is critical for life. While the stress system has been widely studied, the magnitude, and complexity of the various interactions between the its primary components remain elusive (1). Nerve cells in the lateral paraventricular nucleus (PVN), which secrete corticotropin-releasing hormone (CRH) project toward the hindbrain to regions responsible for arousal and sympathetic function. In return, the PVN receives catecholaminergic fibers through an ascending noradrenergic bundle from the locus ceruleus and central sympathetic system. Upon activation, CRH is released into the hypophyseal portal system, which serves as a conduit between the PVN and the CRH neurons with the pituitary, subsequently stimulating adrenocorticotropic hormone (ACTH), and endorphin release by the pro-opiomelanocortin (POMC) neurons of the arcuate nucleus. While the release of CRH and the subsequent stimulation of brainstem arousal and sympathetic centers is part of a positive, reverberating feedback loop, the release of endorphins and ACTH is part of a negative feedback loop that exert inhibitory effects on CRH secretion. ACTH release into the bloodstream acts on the adrenal cortex resulting in the release of cortisol. Cortisol, in turn, exerts negative feedback, both at the level of the pituitary and the hypothalamus (1). Both the acute and chronic activation of the components of the stress system and HPA axis is associated with direct consequences on the activity and functional integrity and of other physiological systems, including those responsible for reproduction, growth, and immunity, which are mostly attributed to the interaction of adrenal hormones with other physiologic systems (1).

The wealth of the available evidence strongly suggests that chronic stress can accelerate aging (2). In addition, however, there is general support that the ability to terminate the stress response systems in the elderly population is impaired (3). It is not clear whether structural and functional alterations in the aging brain, with a commitment decrease glucocorticoid-mediated feedback inhibition contribute to the cortisol hypersecretion observed in the elderly, or whether this is related to functional changes within the adrenal gland itself. The aim of this review is to address adrenal aging with particular focus on alterations in adrenal cortisol production and its implications on stress responsiveness in the elderly.

### Adrenals, Aging, and Stress

#### Aging and Stress

Aging or senescence has served as a focus for research for several decades. While life expectancy has increased significantly, with the age group consisting of individuals over the age of 85 years being the fastest growing age group, our understanding of the aging process remains unknown. Upon critical examination of numerous theories proposed to explain the aging process, two categories emerge that are not mutually exclusive: (1) those that are based on the notion that aging is programmed; and (2) those that are based on the idea that aging is related to the accumulation of damage at a wide gamma of targets and from various sources (4). The cellular senescence/telomere theory supports the idea of a biological clock and suggests that there is a limited replicative life span of normal cells (5). Cell senescence may be triggered in response to stress through different mechanisms, including mutations in signaling, DNA damage from free radicals, or replication (6). Replicative senescence comes from the spoilage of telomeres, resulting after each cell division, n and can be reversed via activation of telomerase, an enzyme that helps regenerate telomeres (7). In stress-induced senescence, the hypothesis is that DNA undergoes alterations due to extrinsic stressors and intrinsic processes, via mutations on the repair enzymes as a result of the dys-functioning and further aging (7, 8). The gene regulation theory of aging supports the notion that genes are responsible for life and death (9). This theory has been supported by findings showing that some genes are responsible for longevity by decreasing insulin-like signaling, and that the life-span could be regulated, in part, by gene expression, similarly to sirtuin, a family of anti-aging genes (9). Other theories, such as the immunological theory, supports the idea that there is deterioration of the normal function of the immune system across aging, with subsequent increased vulnerability to infectious diseases and death (10, 11). The stress theory of aging, sometimes referred to as hormonal theory, supports the notion that the cumulative effects of stress and stressful environments causes disrupts normal cellular function, cause cellular damage, which eventually is expressed in system dysfunction and aging (12). The frequency of stress-related conditions and diseases, such as anxiety disorders, insulin resistance, hypertension, coronary heart disease, depression, cerebrovascular disease, and others, radically increase throughout the lifespan. Additionally, individual differences in vulnerability and resistance to stress and stress-related pathologies may be attributed in part to the heterogeneity of the aging process (13). Primary signaling pathways that respond to stress include the insulin/IGF, TOR, and sirtuin networks (14). Changes in the nutrient grade or the number of stress stimuli result in alterations in these signaling pathways, which alter their mitochondrial function and metabolic activity, via genome proteostasis and maintenance circuits. The network integration and activity of both the stress response system, as well as the maintenance circuitry, which are aimed to augment endurance, develop during the early developmental period. The available evidence suggests that decreased responsiveness and integration of the various components of the stress response, can contribute to both aging and age-related diseases. An important insight in current aging research is that decreased function throughout aging may not be permanent. Rather, it appears that age-related decline can be stunted and the lifespan increased, by increasing the resistance to stress-related processes via conserved signaling pathways (15).

#### Adrenal Glands: Structure and Function

The adrenal gland or suprarenal gland weighs about 5 g consists of two distinct structures, both anatomically and chemically: an inner region, or medulla, that contains catecholamine-producing chromaffin cells and an outer region, or cortex, that is important for synthesizing life-sustaining steroids. The medulla, which produces catecholamines receives sympathetic innervation, while the cortex, which produces life-sustaining steroids is regulated by the pituitary hormones (16).

**Figure 1:**
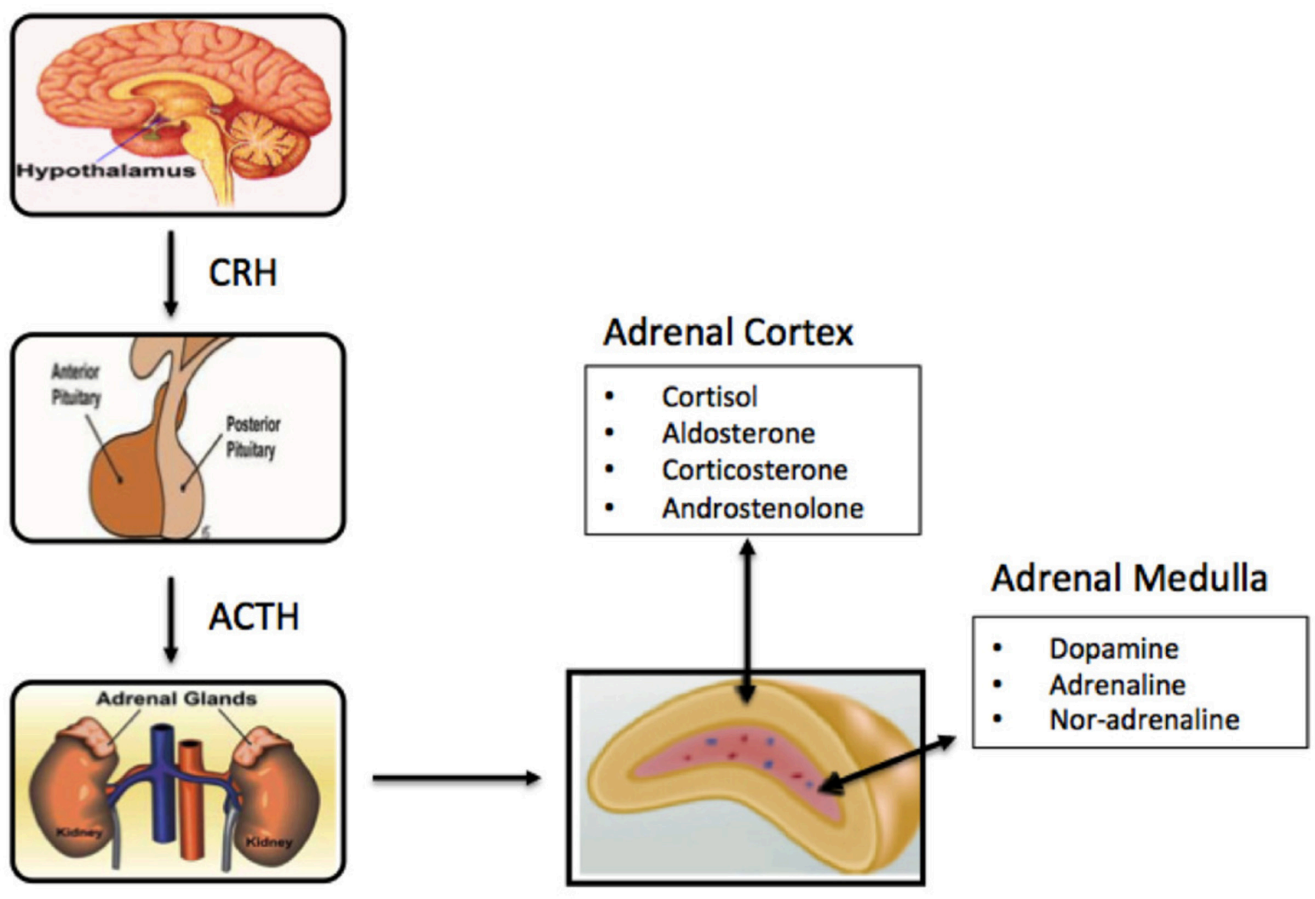
Schematic of components of the primary adrenal axis and main hormones produced in adrenal cortex and adrenal medulla.

The cortex is divided into three zones; zona reticularis (amounting up to 7% of the gland mass), zona glomerulosa (15%), and zona fasciculata (50%), where each of the zones secrete different hormones (Table 1) (Figure 1). All adrenocortical cells contain excessive quantities of lipids, mainly in the outer part of the zona fasciculata. The two inner zones (zona fasciculata and zona reticularis) produce cortisol and sex hormones, including dehydroepiandrosterone (DHEA). Cortisol and its derivatives are known as glucocorticoids due to their function to stimulate gluconeogenesis, raising blood pressure, and regulate inflammation. Due to its latter property, it is often given to patients with systematic inflammatory conditions (e,g., autoimmune disorders), as well as to transplant patients. The outer cortical zone, zona glomerulosa, produces aldosterone in response to the renin-angiotensin system, which regulates body water, and salt. All zones secrete corticosterone, but the actual mechanisms forming cortisol and sex-related hormones are found in the two inner zones, whereas zona glomerulosa has limited aldosterone synthesis (24).

**Table 1:** Hormones of the adrenal glands.

**Adrenal gland**	**Associated hormones**	**Chemical class**	**Main effect**
Cortex: zona glomerulosa	Aldosterone	Mineralocorticoid	Balance water and salt (17)
Cortex: zona fasciculata	Cortisol	Glucocorticoids	Biomolecules (fats, proteins, and carbohydrates) convention to energy (18)
Cortex: zona fasciculata	Corticosterone	Glucocorticoids	Regulate immune response and suppress inflammatory reactions (19)
Cortex: zona reticularis	Androstenolone	Mineralocorticoid	Precursor to male and female sex hormones, testosterone, and estrogen (20)
Adrenal medulla (small amount)	Dopamine	Catecholamines	Regulates pumping strength of the heart and improves blood flow (21)
Medulla: Chromaffin Cells	Adrenaline	Catecholamines	Responds to stress by increasing heart rate (22)
Medulla: Chromaffin cells	Nor-adrenaline	Catecholamines	Vasoconstriction results in high blood pressure (23)

The centrally-located medulla, which constitutes 28% of the gland, is surrounded by the adrenal cortex, and made up of interlacing cords of densely innervated granule-containing cells adjacent to venous sinuses. The cells comprizing the medulla are derived from the nervous system and produce catecholamines (adrenaline, noradrenaline, and dopamine). Stimulation of hormone secretion, leads to release of the hormones into the circulation via exocytosis (25). The medulla of the adrenal gland is considered an important component of the sympathetic nervous system, and houses two primary cell types, the adrenalin-secreting type [90% of cells], and the noradrenaline-secreting type (10%), (13, 16) along with small numbers of sympathetic ganglion cells. While not essential to life, the medulla significantly helps the organism to cope with stress through adrenalin and noradrenalin secretion, which increase the heart rate, convert glycogen to glucose in the liver, among others (26).

#### Adrenal Stress Response: HPA Axis Activation

Exposure to a stressful stimulus results in activation of both the hypothalamic-pituitary-adrenal (HPA) axis and the arousal/sympathetic system, which comprise primary components, the central and peripheral parts, of the stress system. Of the variety of factors that are produced and released in the stress response, the mediators of the HPA axis, particularly the glucocorticoids, are critical (1). Normally, after exposure to a stressor, glucocorticoids act on the brain to restore physiological, and behavior homeostasis. Glucocorticoids produce adaptive responses by exerting effects on various central and peripheral sites, in addition to exerting effects on wide span of neuronal activities, such as nerve cell excitability, neuroplasticity, neurogenesis, neuronal death, stress responsiveness, and behavioral responses. The glucocorticoid, namely via cortisol, negative-feedback loop comprises a critical part of the adrenal stress response as it acts to terminate HPA activation. The adrenal steroids appear to exert their effect via the interaction with intracellular receptors that show specific, and high affinity ligand binding. Two types of receptors for adrenal steroids have been identified in the brain and the pituitary (27). Both glucocorticoid receptors have been found in the brain and have been implicated in basal and stress-associated negative feedback control of the HPA axis. The type I, or mineralocorticoid (MR), receptor appears to mediate, and regulate the tonic influences of glucocorticoids on brain functions at basal levels. Activation of the type II, or glucocorticoid (GR), receptor plays an important role in blunting further activity of the stress response through negative feedback suppression of the stress response. Changes in learning and memory, as well as increased anxiety is associated with activation of GR. These functional changes are anatomically encoded within distinct neural regions and structures. The hippocampus (HC) and prefrontal cortex (PFC) are largely inhibitory of the limbic-HPA axis activity, and the amygdala appears to activate the stress response. Elevated levels of glucocorticoids appear to impair synaptic plasticity in the HC and the acquisition of HC-dependent memories. GR and MR are both abundantly expressed in neurons of the HC, PFC, and amygdala. MRs and GRs may have opposing functions in regulating hippocampal synaptic neuroplasticity during the stress-response. Activation of MRs may be a prerequisite for hippocampal plasticity, while GRs may exert an inhibitory effect on plasticity (1, 28–30).

#### Adrenal Gland Changes With Aging

As physiologic functions gradually decline during aging, a reduction in activity across the hypothalamic-pituitary-adrenal (HPA) axis occurs. The HPA axis is fundamental to homeostasis, acting as a regulator of stress response (31). During the multifactorial process of aging, the secretory pattern of the adrenals, especially of the adrenal cortex, is subject to quantitative and qualitative alterations, and so is the axis's negative feedback sensitivity to the end hormones (32), probably contributing to the pathogenesis of age-related disorders, particularly the decline in cognition observed in older people (33). In the aging population, several studies revealed an improved physical and cognitive performance during higher activity of the HPA axis, compared with reduced activity of the axis (34, 35).

### Adrenal Hormone Alterations During Aging

It has been suggested that aging is related to the loss of balance between the two fundamental process, damage, and repair (36), as well as tissue/organ loss over time (37, 38). This natural gradual deterioration of function is modulated by the stress system and weakening of the normal pathways of repair, such as DNA damage repair, mitochondrial metabolism, and proteostasis. In humans, aging is characterized by an increase in adrenal glucocorticoid secretion and a decrease in adrenal androgen synthesis. As aging occurs, several changes in hormone levels taking place.

The cortisol secretion pattern by zona fasciculata of the adrenal cortex undergoes several modifications with age. Unlike most hormones whose levels diminish throughout aging, mean cortisol concentrations increase (39), displaying generally irregular patterns and a flattened circadian profile (40, 41), an evening and night time higher nadir (33, 39), and an attenuated awakening response with an earlier morning level peak (32). Additionally while aging, there is diminished negative feedback on the secretion of cortisol, due to impaired sensitivity of the HPA axis (33, 42). This age-related attenuation of axis negative feedback may be associated with several factors, such as vascular components, reduced number of brain glucocorticoid receptors, differences of cortisol concentration in the cerebrospinal fluid (CSF), and alterations of cortisol clearance in the blood brain barrier or the CSF (42).

Increased cortisol levels and diminished axis sensitivity are generally related with inferior cognitive status, dementia of degenerative and vascular cause (43), depression, and anxiety (39). Furthermore, higher urinary free cortisol concentrations are associated with Alzheimer's disease (44) and increased salivary cortisol concentrations in older people are associated with increased mortality risk, higher risk of diabetes mellitus, and hypertension (45). Additionally, 11-β hydroxysteroid dehydrogenase, which acts to transform cortisone into active cortisol, shows increased activity during aging, affecting tissue cortisol availability (46).

Frailty has also been associated with elevated diurnal cortisol levels (47, 48), a state of increased vulnerability of the aging population. As a catabolic hormone, higher cortisol levels are linked with characteristic clinical features of frailty such as weight loss, muscle mass reduction, and anorexia (49). On the contrary, lower diurnal cortisol levels are associated with longevity (50).

Dehydroepiandrosterone (DHEA) and its sulfate ester (DHEAS), produced and secreted by zona reticularis of the adrenal cortex in response to ACTH stimulation, decrease profoundly during aging (39). Adrenal secretion of DHEA gradually declines over time at a rate of 1 ± 2% per year (42), constituting one of the biggest endocrine changes found in human aging, with a 5- to 10-fold decrease (51) resulting in “adrenopause” (52). By the age of 70–80 years, DHEAS are about 30% of peak values in women and 20% in men, compared with people under 40 years of age (32, 53). In peripheral tissues, DHEA/DHEAS convert into androgens and oestrogens, posing a significant role, especially in older men, where <50% of these androgens are produced from the testicles (32).

DHEA/DHEAS secretion is considered of great significance in frailty (49). Higher levels have been linked with improved health outcomes (51), improved psychological status and functional abilities, muscle strength, higher bone density, anti-inflammatory actions (54), reduced risk of death from cardiovascular disease (55), and increased longevity in males (56). Many cross-sectional studies have found correlation between several diseases (e.g., Alzheimer's disease, type 2 diabetes, and depression) and DHEA-S levels (31). Lower DHEAS levels have been associated with deficient mental health (39), as well as increased cardiovascular mortality and cardiovascular events in people aged over 50 (54).

The reduction in DHEAS levels with the simultaneous preservation of plasma cortisol, reveal a dissociation of the cortical secretory pattern, which may be caused by selective depletion in zona reticularis cells leading to impairment of androgens, rather than being controlled by a hypothalamic aging pacemaker (42, 52). In particular, zona reticularis cells seem to be susceptible to vascular injury and possibly to the intra-adrenal gradient of autocrine and paracrine elements, leading to cell damage (42). Additionally, the response of DHEA to exogenous ACTH administration is notably diminished with age (57).

The concentration ratio of glucocorticoids to DHEAS is closely tied to aging, with a gradual increase. Cortisol has neurotoxic effects by stimulating neuronal degeneration through increased susceptibility to metabolic and vascular injuries, reduction of dendritic length, and cell death possibly associated with apoptosis (33). On the other hand, DHEAS enhances long-term potentiation of neurons and protects from structural damage and functional impairment, promoting glial, and neuronal survival (42). Consequently, the observed increase in the cortisol/DHEAS ratio during aging, leads to enhanced neurotoxicity and probably contributes to the occurrence of age-related neurodegenerative illnesses.

Aldosterone secretion and release from the adrenal cortex declines with aging (58). Basal levels of aldosterone decrease (51, 59), with an associated reduction in renin activity. This characteristic age-related decline in plasma aldosterone refers to men and women as well (60). Despite the limited number of studies and small samples in most of them, the common observation of decreased aldosterone secretion and plasma renin activity in elders, may have significant effects on various aspects related to evaluation and treatment of hypertension in old individuals (58, 61).

Regarding adrenal medullary function, basal adrenaline secretion decreases with age (62). Epinephrine and norepinephrine plasma concentrations become lower or don't change significantly with advancing age (63, 64), so lower secretion from the adrenal medulla in older people is not apparent from plasma concentrations, mainly because of the reduced clearance of these hormones from the circulation (62). Additionally, in cases of acute stress, epinephrine release is mainly lessened in older people, and stimulable elevation in serum catecholamines (as percentages of basal values) also decrease (65, 66). In one study, adrenaline production from the medulla was lower by 40% in elderly healthy men, compared to younger healthy men. Furthermore, adrenaline release in response to stress was increased 33–44% in older men of that observed in young controls. The exact mechanisms responsible for the decrease in adrenaline release from the adrenal medulla observed with aging, have not been fully verified. To some extent, they are possibly related to an age-related decrease in pre-ganglionic nerve activity, reduction in response to pre-ganglionic nerve activity in the adrenal medulla, or possibly depletion in adrenaline synthesis, and storage to the adrenal medulla (62). Conclusively, current evidence shows that adrenal medullary secretion and release of epinephrine are lower in older people, both at rest and during stress (67).

**Figure 2:**
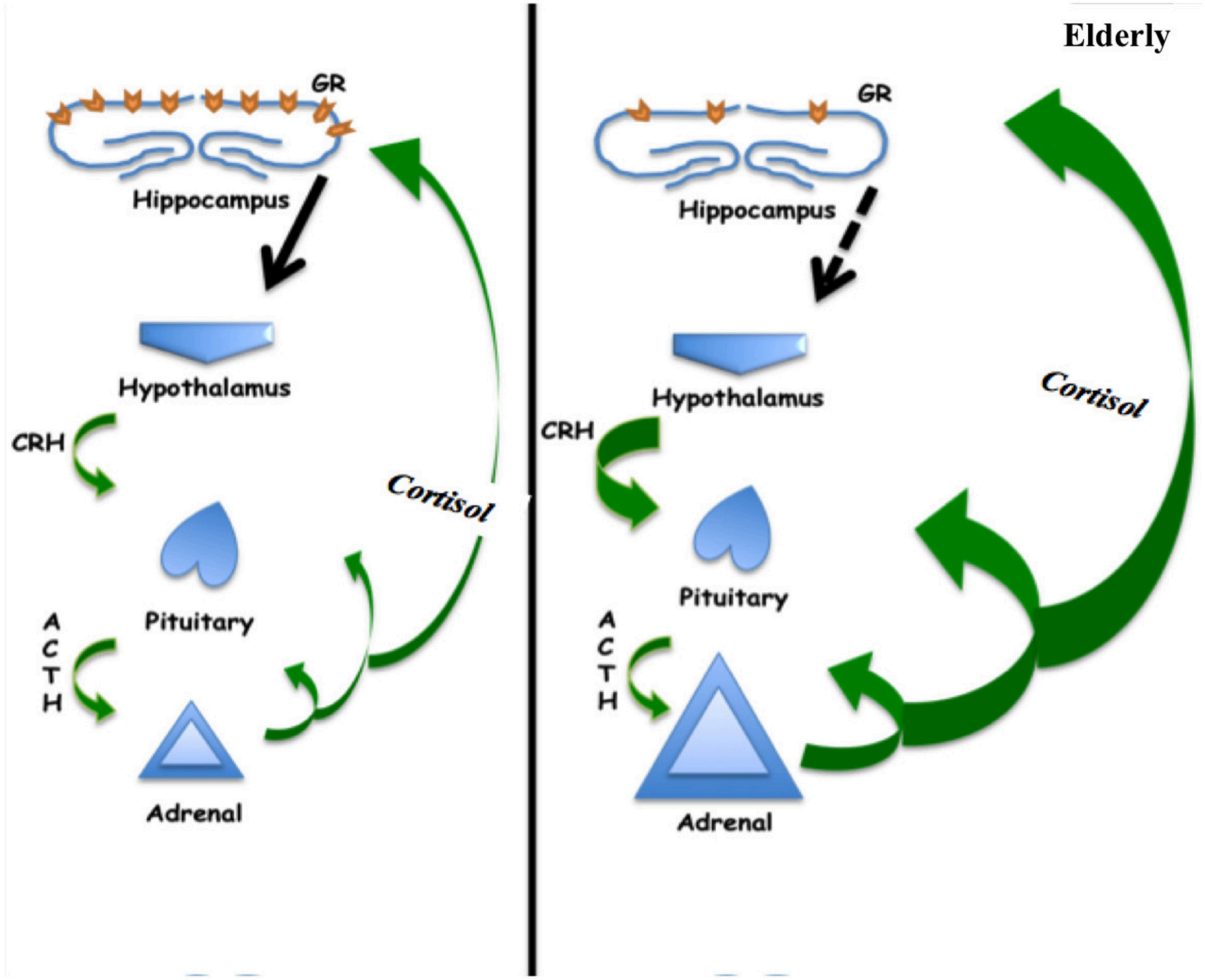
Schematic of hypothalamic-pituitary-adrenal axis showing increased cortisol production in the elderly, which may be associated with decreased negative feedback at the hippocampus related to decreased glucocorticoid receptor concentration.

The circadian rhythm is regulated by the hormone melatonin which shows a decrease in levels throughout aging (68). The decrease in melatonin concentrations has been associated with increased incidence of disruption of the normal circadian rhythm in older adults (69). Melatonin is also known to have an immunomodulatory role. While a functional restructuring of activity of the immune system is an integral part of aging, it is not clear if this is associated to changing levels of melatonin (70). Additionally, as a potent antioxidant, melatonin was reported augment cardiovascular function, mostly by its hypotensive effects (71). This notion is further supported in a study on individuals with non-insulin dependent diabetes mellitus, where supplementation with melatonin was found to improve antioxidative defense (72). Of note, melatonin administration improved the circadian rhythm, including sleep and activity at night, but produced no notable changes on daytime activity and naps in Alzheimer type of dementia (73). Finally it has been suggested that melatonin may serve to protect elderly from delirium when given at low doses during acute care (74) (Figure 2).

### Association of Adrenal Aging on Other Systems

**Figure 3:**
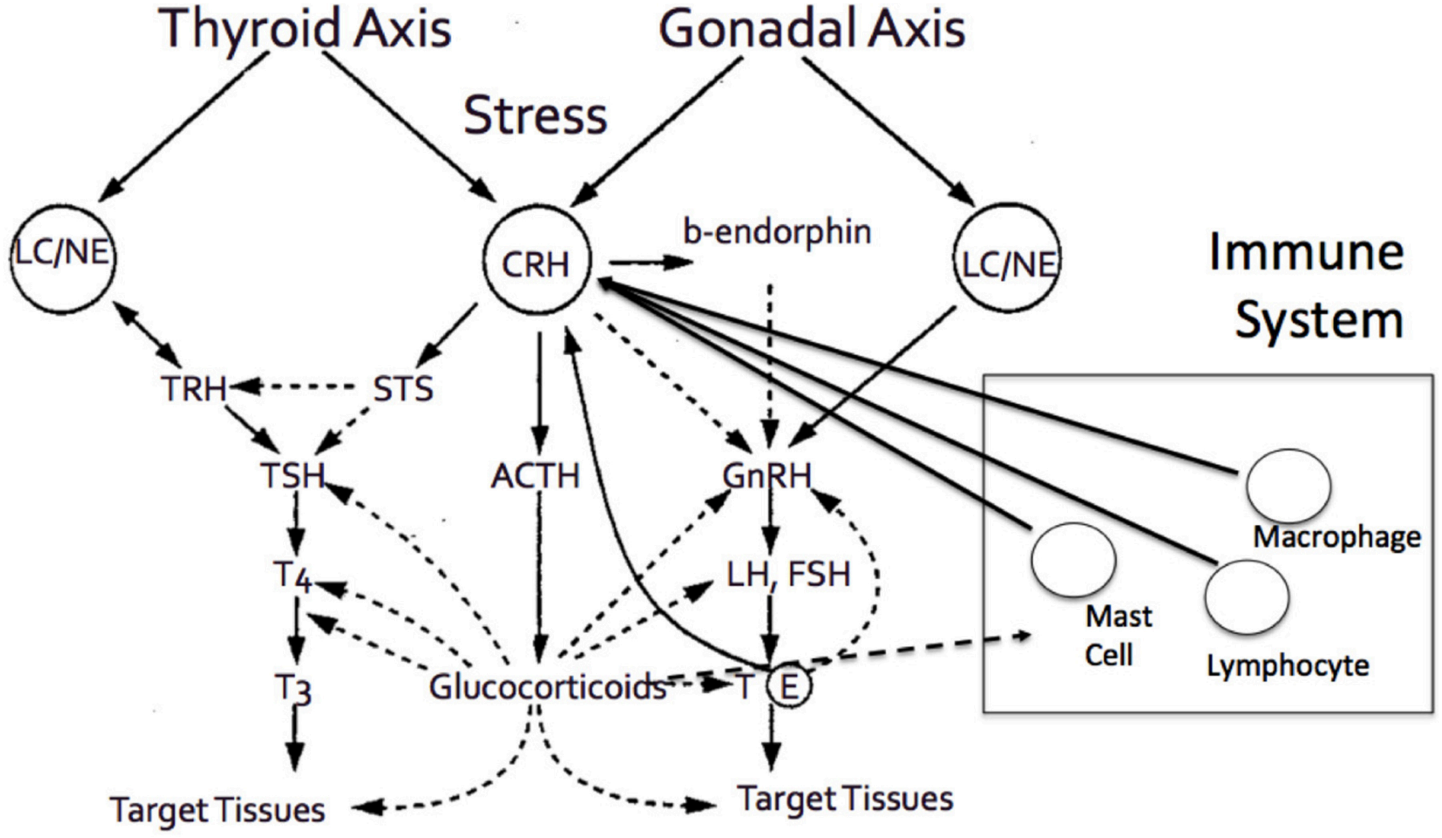
The adrenal axis and the end effector, cortisol, demonstrates tight interactions with various other hormonal axes, and systems, including thyroid axes, gonadal axis, and immune system, among others.

Aging involves a gradual decline in all human functions, including adrenal deterioration. Inevitable clinical sequelae include alterations in body composition, such as loss of density of bone minerals, muscle mass loss, and fat mass increase. These changes may also be related to the endocrine system adjustment to aging (52). Specifically throughout aging, the increase of cortisol levels can cause various effects on multiple systems and adverse changes in older people (Figure 3). As previously stated, elevated cortisol availability has been associated with significant body alterations, leading to the fundamental characteristics of frailty and other functional abnormalities (49). Additionally, while aging, the activity of type 1, 11β-hydroxysteroid dehydrogenase is enhanced in various tissues, such as the central nervous system, skeletal muscles, bones, and skin (46, 75, 76), leading to increased local cortisol formation. This may be clinically correlated with cognitive decline, sarcopenia, osteopenia or osteoporosis, and skin atrophy. Some of the most prominent clinical manifestations of adrenal aging and cortisol increase are briefly discussed below.

#### Visceral Obesity and Loss of Muscle Mass

Certain characteristic changes in body composition are observed in older persons. These include a decline in total body weight, gradual loss of fat mass (which is normally increasing until the age of about 65), loss of muscle mass, and accumulation of visceral fat (60, 77). Cumulatively, these changes lead to higher total body fat mass and lower total lean mass. Endocrine changes reflected in these alterations include the aforementioned increase in cortisol levels (which is also in part due to the increased production of cortisol by the adipose tissue), insulin resistance, and decline of serum testosterone (32, 78, 79). Total muscle mass reduces by ~30% by the age of 80. This is widely known as sarcopenia, the age-associated loss of skeletal muscle mass and function (79), a phenomenon with important healthcare, and socioeconomic implications. In particular, previous studies have associated muscle loss and fat accumulation with increased urine cortisol secretion (80) and have shown that this decrease of muscle mass and strength is in part due to lipid infiltration of the muscle, resulting in change of muscle quality (81).

#### Diabetes Mellitus

During the aging process, significant changes of glucose homeostasis include lower levels of insulin and gradually increased resistance to its action (31). Total body composition changes that accompany aging, also promote susceptibility of older people in developing diabetes, by augmenting insulin resistance. As previously mentioned, increase in visceral fat, obesity and alterations in fat to lean muscle mass ratio, affect insulin action, contributing to diabetes pathogenesis in older people (82, 83). In addition, islet β-cells undergo quantitative and qualitative dysfunction, consequently affecting insulin secretion, which is independent of peripheral tissue resistance (84). In fact, in older individuals, β-cell deterioration has a more significant role in the development of diabetes compared to younger adults (32).

Cortisol as a catabolic hormone significantly affects glucose metabolism. Higher cortisol concentrations are associated with insulin resistance and increased fasting glucose (85). It was also demonstrated that the risk of developing diabetes increases with elevated cortisol levels in older people (45). Furthermore, a flatter diurnal slope of cortisol profile (a pattern found in older adults) is related with type 2 diabetes (86).

#### Osteopenia

One of the most apparent and inescapable effects of aging is a decline in bone mineral density, leading to osteopenia, osteoporosis, and increased risk of fractures. Bone density increases until adulthood, followed by a stable period and thereafter a gradual age-related decline (77). Advancing age impairs bone structure because of an imbalance between bone formation caused by osteoblasts, and bone reabsorption by osteoclasts. Excess of cortisol during aging contributes to the inhibition of bone formation, through stimulation of osteoblast and osteocyte apoptosis (87), extension of osteoclast survival, and suppression of new osteoblast formulation (32). Bone cell glucocorticoid receptors seem to pose an important role to the negative impact of elevated cortisol levels on bone metabolism (88).

#### Immune Function

Most body systems and organs, including that responsible for immune function, undergo slow, and continuous changes throughout the aging process that ultimately compromises their normal function (89). Among the various factors that change throughout aging and serve critical roles in immunosenescence are a altered capacity for cytotoxicity of natural killer cells, atrophy of the thymus, decreased neutrophil function, reduced number of naive T cells, as well as decreased B cells antibody production (90). It is noteworthy that the HPA axis or stress axis has a critical role in immune system function modulation.

While both adrenal hormones, DHEA and cortisol, modulate immune function, they have opposing effects. Cortisol plays an important role in immunosuppression, while DHEA enhances immune function (89). The immune-enhancement properties of DHEA is associated with changes in its production, which begin decrease after puberty reaching almost 5% circulating concentrations in the elderly compared to pre-puberty (90). On the other hand, cortisol levels remain unaltered, a fact that leads to an imbalance between the two stress hormones (89). The evidence suggests that DHEA increases mitogen-stimulated IL2 release from CD4^+^ cells and this counters the changes in CD8^+^ produced by glucocorticoids (91). This suggests that an increase in the ratio cortisol:DHEA may contribute to the decline in immune function observed in the elderly. Thus, DHEA supplementation in the elderly may provide beneficial effects to immune function (92). In addition, stress management as well as acute exercise seem to slow immunosenescence as they improve the cortisol:DHEA ratio (93).

There is ample evidence showing that the effectors of the HPA axis, particularly glucocorticoids, can influence immune function, and immunocompetence via various mechanisms (1). While the data remains conflicting, in general, the elevated levels of circulating cortisol achieved during chronic stress or aging exert immunosuppressive and anti-inflammatory effects. Glucocorticoids do not always suppress immune function, but rather they may act to increase aspects of immune function. None-the-less, hypercortisolemia is associated with augmented function of suppressor T-cells, reduced leukocyte traffic, diminished normal cell-mediated immunity, decreased cytokine production and function, lymphopenia, loss of normal lymph node mass, and thymic involution (94).

### Adrenal Aging and Brain Function and Behavior

One of the key questions in neurobiology is how stressful experiences across the lifespan alter the aging process and influences vulnerability to dysregulation of the normal stress response. States of stress induced by psychosocial factors can result in deleterious effects upon the well-being of individuals and predisposing to a variety of disorders. Chronologic age is also a significant predictor of chronic diseases. Psychological stress appears to be a critical aspect in promoting biological aging and earlier onset of age-related disease.

The hippocampus (HC), prefrontal cortex (PFC), and amygdala (AMYG) are highly interconnected key brain regions implicated in stress. Stress induces profound behavioral changes that are paralleled by structural and plastic changes in these areas. HC serves as an important connection between the cortex and hypothalamus, regulating in part, cortisol diurnal rhythm. The HC has an overall inhibitory effect HPA axis activity, serves as a primary central target of stress hormones, and is extraordinarily vulnerable to stress. A key function of the PFC includes the transient storage and manipulation of information to guide subsequent behavior. Dysfunction of the PFC is noteworthy in several psychiatric disorders. The dorsolateral PFC (DLPFC) is important in the conscious regulation of emotion to reduce fear responses and is involved in negative feedback HPA axis regulation. The medial (m) PFC has been implicated in the pathogenesis of MD and SZ and influences HPA axis activity. It has a central role in regulating emotions, reward encoding, and goal directed learning. The mPFC is tightly connected with the DLPFC and limbic areas, particularly the AMYG, which has a central role in the detection of threat and fear. In contrast to the HC and PFC, which decrease in volume after chronic stress, the AMYG increases, which is associated with enhanced anxiety. During emotional challange, the PFC exerts control over the AMYG; successful emotion regulation is associated with increased PFC activity and decreased AMYG activity (28–30).

Stress is a risk factor that affects the physical, mental and social health of individuals through lifespan (95, 96). It is associated with aging-related outcomes at cognitive, emotional, mental, and neurobiological level (97). Over the past decades, there has been an increased research focus on stress and stress mechanisms worldwide due to the aging population and the high morbidity associated with stress-related diseases. Evidence suggests that there is an interplay between chronic stress and the development of depression, anxiety, insulin resistance, dementia as well as cardiovascular diseases (97, 98). Although there is ample evidence about the role of stress in chronic diseases; however the relationship of human biology and environmental factors in terms of causality, remains unclear. It is not feasible to ascertain whether the neurobiological alterations lead to stress-related health outcomes or the environmental stress-related factors result to higher stress levels and neurobiological variations. In other words, cortisol levels are affected by both environmental and endogenous factors.

Aging is accompanied with decrease of and deficiencies in autonomy, health, and social status which entail elevated stress (28). There is a heightened emphasis of the role of the HPA axis in aging and its subsequent effects on the stress-adaptability, stress resistance, and stress-related pathologies (41). The role of HPA axis in stress-related pathologies is well-established mainly due to its sensitivity in both chronic and acute stress, though neurophysiologic variations do exist among individuals and result to differences in aging process, vulnerability, resilience, and stress regulation (41). The variations of the HPA axis by age are in line with the different aging pathways and sub-groups identified in the general population but there is no substantial evidence to determine the consistency of this relationship. Some researchers suggest that older adults experience an anticipated decline in terms of health status which is accompanied by declined cortisol levels (99, 100). On the contrary, according to other studies cortisol levels increased by age (101, 102) while others support that there is no association between cortisol levels and aging (103). There is also the case of elderly that maintain a high level of health status and a normal HPA axis function but they cannot be considered as a representative group of the general population, as well as the elderly chronic patients with poor health status and the most significant deterioration in HPA axis function (41).

Notwithstanding the correlational and not causal relationship between stress, HPA axis and aging, evidence revealed that age-related HPA axis changes affecting the health outcomes of older adults mainly via the diurnal cortisol secretion pathway (78). Elevated adrenal glucocorticoid levels associated with chronic stress have been implicated in alterations in spatial memory, hippocampal function, and cognitive status, in general (104). Negative or traumatic experiences earlier in life, shape the diurinal pattern of cortisol and indicate an individual's level of exposure to chronic stress and subsequently the predisposition for depression, anxiety, and other chronic diseases (41). HPA hyperactivity is linked to higher anxiety levels and increased depressive symptoms. Decreased DHEA and dehydroepiandrosterone sulfate (DHEA-S) release are often found in patients with major depressive disorder (105, 106) while increased DHEA-S is associated with aggressive behavior (13, 107). Furthermore, resilience constitutes a case in point of the interplay between endogenous and environmental stress-related factors and aging and thus it can be used to map the trajectory of HPA axis, stress, and aging (108). Resilience is strongly associated to emotion regulation and social resources (e.g., social support) which in turn affects the HPA axis functioning and vice versa (108, 109). Higher diurinal cortisol levels have been identified in people with low social support and poor resilience which in turn is associated with increased risk for chronic disease and multiple bio-psychosocial implications (41). A healthy aging of brain function is closely related to the quality of health across the life-span and facilitates normal behavior and society integration. The evidence supports that the early prenatal environment has a tremendous impact on later brain aging. Moreover, these early environmental effects in addition to life-style and genetic constructs can have notable effects on age-related brain disorders. Therefore, at health policy context, it is important to develop interventions and programs with the aim to strengthen protective factors such as social support in older adults, so as to increase emotional regulation, reinforce resilience, and decrease the HPA axis dysregulation. Both the aging process, as well as chronic stress have been associated with altered brain function, with consequences in cognitive and emotional processing and an increased vulnerability for brain disorders. Regionally specific changes in brain structure and function associated with chronic stress and aging is associated with increased depression, cognitive changes, anxiety, among others.

### Adrenal Aging: Response to Injury and Surgical Stress

Trauma and injury are well-known factors of homeostasis disruption that cause stress to living organisms. Surgical trauma is a controlled and standardized injury in the sterile environment of the operating theater on a patient receiving pharmacologic treatment for pain control with or without anesthesia. Despite this, surgery is a major stressor causing an inflammatory reaction with activation of numerous cytokines, mobilization of cellular response, and a well-defined hormonal response (1). The two most studied systems controlling the injury and stress response are the HPA axis and the sympathetic/parasympathetic autonomic nervous system (2). Mediators, such as pain, anxiety, cholecystokinin, angiotensin II, vasopressin, vasoactive intestinal polypeptide, catecholamines, and proinflammatory cytokines stimulate the secretion of hypothalamic CRH. CRH stimulates the release of ACTH from the anterior pituitary, which in turn stimulates glucocorticoid synthesis and secretion from the zona fasciculata of the adrenal cortex (110). Glucocorticoids are synthetized from a cholesterol moiety and they diffuse readily through the cell membranes to reach the cytosol glucocorticoid receptor of target cells in almost every tissue of the human body. Steroid receptors are inactive by forming a complex with several different molecules of heat shock proteins. Binding of the glucocorticoid molecule to the steroid receptor unbinds the heat shock protein and allows the complex to enter the nucleus where it induces DNA transcription and protein synthesis (111).

#### Effect of Surgery on the HPA Axis

Researchers have discovered from the early eighties that surgery produces changes in the cortisol circadian rhythm. McIntosh et al demonstrated that in a small group of 10 patients, serum cortisol levels had significantly increased in the second postoperative day after upper abdominal surgery. They also found that this increase was influenced by the type of surgery; high trauma surgery patients had two times greater increase of their serum cortisol levels on postoperative day two in comparison to low trauma patients (112). Ten years later, Naito et al investigated the alterations of the HPA axis in patients undergoing major upper abdominal surgeries such as total gastrectomy, pancreatoduodenectomy, and colectomy. All patients presented a prompt and marked intraoperative elevation of plasma CRH, ACTH, and cortisol levels. Interestingly, both CRH and ACTH had a biphasic change; after this initial peak, they decreased during the first postoperative days to 50% of the preoperative values and returned to normal by postoperative day seven. Intraoperative plasma cortisol levels were more than two times higher than the preoperative levels and progressively dropped down to normal values by postoperative day seven (113). Pooling results from several studies, patients undergoing major surgery present a peak of serum cortisol concentrations from 30 to 45 μg/dL (114). Newer clinical studies compare laparoscopic to open cholecystectomy and laparoscopic to open Niessen fundoplication procedures. Two randomized controlled trials and one prospective study, report that the laparoscopic procedures reduce the acute phase component of surgical injury expressed by serum interleukin 6 (IL-6), C-reactive protein, and prealbumin but do not attenuate the hormonal response expressed by serum cortisol levels (115–117). A study by Siekmann et al. assessing the inflammatory response of patients undergoing colorectal surgery similarly reported that the median serum cytokine concentration of IL-6, IL-8 and IL-10 at one to 6 h after surgery in patients undergoing open surgery was higher when compared to laparoscopic surgery (118). Similarly, a randomized controlled trial by Veenhof et al. found that 2 h after laparoscopic colectomy HLA-DR expression on monocytes was significantly higher and IL-6 level increase was significantly lower compared to open colectomy. However, no difference in serum cortisol levels was evident between the two techniques in both studies (118, 119). Given the diurnal variation of cortisol and the pulsatile secretion of CRH and ACTH it should not be surprising that studies not specifically aiming to investigate the HPA axis may be underpowered to demonstrate differences in postoperative cortisol levels between open to laparoscopic techniques. There is sufficient evidence to support that the extent of surgical trauma influences the secretion of CRH, ACTH, and cortisol during the intraoperative and early postoperative period. Surgery causes, from the moment of the surgical incision, a marked increase in serum CRH, ACTH, and cortisol with all three hormones dropping to normal levels during the early postoperative period.

#### The HPA Axis of Elderly Patients Undergoing Major Surgical Procedures

In general, basal ACTH secretion, as well as basal and stimulated, cortisol release does not change in the elderly (120). Regardless of age, the cortisol levels are similar in patients with acute myocardial infarction and ACTH stimulation tests showed no difference in cortisol peaks (121). Historic data from autopsy series found that the adenohypophysis is subjected morphological changes in old age undergoing weight reduction and fibrous shrinkage, however, these changes do not correspond to functional degradation (122). Contradictory data come from a Japanese retrospective study of 96 patients with large symptomatic pituitary tumors. Patients over 70 years of age suffered more frequently acute adrenal insufficiency and severe hyponatremia in comparison to younger patients with the authors suggesting that the HPA axis functional reserve is reduced by old age (123).

Old age is not synonymous with incapacity and frailty. Frailty is associated with an increased vulnerability to stressful stimuli, with a decreased ability to maintain a controlled, normal response to intrinsic and environmental stressors, and decreased ability to maintain both physiological, and behavioral homeostasis. Almost 20–30% of the population over 75 years of age is associated with geriatric frailty, which increases notably with advancing age (124). Empirical knowledge dictates that older patients recover slower after major surgical procedures. A large metanalysis of 5,186 patients analyzing surgical outcomes following pancreaticoduodenectomy in elderly patients reported increased post-operative mortality and pneumonia in patients over the age of 75 years, and increased post-operative complications in patients over the age of 80 years (125). Watters et al investigated the recovery of strength in patients older than 70 years of age after major abdominal surgical procedures when compared with patients younger than 50 years of age. Older patients had lower preoperative strength, lower absolute postoperative strength levels, less rapid, and less complete recovery of strength. However, postoperative urine cortisol levels were similar in old and young patients (126). Considering the previous studies older people should not be at an increased risk of adrenal failure. Nevertheless, data from a nationwide study in Taiwan reports that adrenal insufficiency has an incidence in individuals over 60 years old is 92.4/10^5^ of the geriatric population that is six times greater than that observed in the general population. Most of these patients have severe comorbidities, infectious and pulmonary diseases, fluid and electrolyte disorders, and complicated diabetes mellitus (127). Sepsis/SIRS, various drugs, HIV, CMV, and systemic fungal infections are well-known causes of primary adrenal insufficiency (114). A plausible explanation may be that older patients may have a reduced HPA functional capacity due to subclinical secondary adrenal insufficiency from the comorbidities of old age and not the aging process of the adrenal glands.

## Conclusion

Both normal aging and chronic stress appear to affect the body via shared mechanisms related to glucocorticoid function. The chronicity of both the aging process, particular in relation to alterations in the structure and function of the adrenal gland, and stress can be detrimental to an individual's general well-being. The available evidence supports that the synergy of aging and chronic stress, via their common end-point effector cortisol, can adversely affect the function of numerous vital systems, leading to neural and cognitive changes, osteopenia, diabetes mellitus, visceral obesity, altered immunocompetence, among others.

## Author Contributions

All authors listed have made a substantial, direct and intellectual contribution to the work, and approved it for publication.

### Conflict of Interest Statement

The authors declare that the research was conducted in the absence of any commercial or financial relationships that could be construed as a potential conflict of interest.
